# Study protocol of a randomised controlled feasibility study of food-related computerised attention training versus mindfulness training and waiting-list control for adults with overweight or obesity

**DOI:** 10.1186/s13063-019-3932-0

**Published:** 2020-01-10

**Authors:** Daniela Mercado, Jessica Werthmann, Iain C. Campbell, Ulrike Schmidt

**Affiliations:** 1 Section of Eating Disorders, Department of Psychological Medicine, Institute of Psychiatry, Psychology and Neuroscience, King’s College London, De Crespigny Park, London, SE5 8AF UK; 2 Department of Clinical Psychology and Psychotherapy, Institute of Psychology, Albert-Ludwigs University of Freiburg, Freiburg im Breisgau, Germany; 3 South London and Maudsley NHS Foundation Trust, London, UK

**Keywords:** Obesity, Attention trainings, Mindfulness, Attention bias, Trial

## Abstract

**Background:**

Obesity is a highly prevalent condition with multiple adverse health consequences. Widely available first-line treatments for obesity, such as dietary and other lifestyle interventions, typically have only short-term effects. Thus, new treatment approaches are needed. Novel interventions such as Attention Bias Modification Training (ABMT) and mindfulness-based interventions focus on modifying different maladaptive cognitive patterns typically present in people with obesity (e.g. attention bias to food cues); however, their mechanisms of action remain largely unknown. We describe the theoretical basis and the rationale for a study protocol of a feasibility randomised controlled trial (RCT) comparing two attention trainings (ABMT vs Mindfulness Training [MT]) in people with overweight or obesity. The aim of this study is to inform the development of a large-scale RCT in relation to acceptability and attendance rates and to identify preliminary evidence for the interventions’ clinical efficacy and potential underlying mechanisms.

**Design:**

Forty-five adults who are either overweight or obese (minimum body mass index of 25 kg/m^2^) will be randomly allocated to receive eight sessions over eight weeks of either computerised ABMT or MT or be on a waiting list. Clinical and cognitive outcomes will be assessed at baseline, post-treatment (8 weeks) and follow-up (12 weeks post-randomisation). These include mood, body composition and attention biases. Credibility and acceptability of the trainings will be assessed using questionnaires, and recruitment and retention rates will be recorded.

**Discussion:**

Findings will inform the feasibility of developing a large-scale RCT that takes into consideration effect sizes for primary outcome measures and the acceptability of the design. The study will also provide preliminary evidence on the clinical efficacy of two different attention trainings for people with obesity and associated underlying mechanisms.

**Trial registration:**

ISRCTN Registry, ISRCTN15745838. Registered on 22 May 2018.

## Background

Obesity is a highly prevalent condition in the western world, associated with multiple adverse health consequences such as diabetes mellitus, hypertension and some types of cancer [1, 2]. Current treatments for obesity, based on dieting and lifestyle changes, typically have only short-term effects [3–6], presumably because there is little focus on pre-conscious and relatively automatic cognitive processes that may drive overeating in people with obesity [7].

Several studies have investigated the role of reward and of attention control in relation to food in people with obesity and/or binge eating disorder (BED), an eating disorder leading to (and often co-morbid with) obesity. These studies show enhanced reward sensitivity when participants are presented with food cues (both neurally and self-reported) and lower self-regulatory processing [8, 9]. Relatedly, some studies have reported the presence of attention biases to food and other salient cues in obesity and BED [10, 11] presumably due to low attention control for highly rewarding stimuli (e.g. high-caloric food) [12–14].

Attention biases (AB) occur when salient stimuli (e.g. food) capture a person’s attention in comparison to neutral stimuli [15]. Research suggests that people with obesity and/or with BED tend to have AB towards food cues together with a difficulty in disengaging from these cues (as measured with eye-tracking techniques) [11]. Importantly, gaze maintenance on food cues has been shown to contribute to food craving and to subsequent food consumption [10, 16] and AB has been positively associated with body mass index (BMI) [17]. Due to the potential role of AB in eating behaviour, treatment approaches aimed at modifying these biases have been developed [18, 19].

It has been proposed that AB can be targeted directly, using attention bias modification training (ABMT), a form of cognitive bias modification training. Specifically, ABMT has the potential for modifying attention processes towards salient cues [20]. These types of cognitive trainings hold the promise of an accessible treatment option due to their low cost and their potential for self-administration. Moreover, cognitive bias modification trainings, such as ABMT, target specific relatively automatic cognitive processes directly and do not, as in the case of cognitive behavioural therapy (CBT), rely on the patient’s ability to consciously modify maladaptive pre-conscious cognitive processes such as AB.

In the context of overeating, there is a growing body of research on the effects of ABMT. Some studies have focussed on using ABMT experimentally to investigate the potential (causal) role of cognitive processes associated with the onset/maintenance of eating and weight disorders [21]. Others have started to test the clinical impact of ABMT as treatment for overeating tendencies. In these studies, different ABMT paradigms have been used, but the most commonly investigated is the dot-probe task [22].

Even though results have been mixed, some studies using ABMT to modify food-related AB and eating behaviour have shown promising results. One meta-analysis reported a reduction in high-calorie food consumption (medium effect size) after training participants to look away from high-caloric food cues [23]. A second meta-analysis of the effects of different cognitive bias modification trainings (including ABMT) reported a medium effect size in modifying AB towards food cues in individuals with normal weight and with overweight/obesity [24].

In addition, a study using a single session of ABMT to train people with BED to look away from food cues found a significant reduction in subjective food craving after training [25]. Relatedly, a feasibility open trial using eight weekly sessions of ABMT in people with overweight/obesity who binge eat reported encouraging results in reducing weight, eating disorder symptoms, binge eating and AB after training [26]. To date, however, the mechanisms involved in the potential therapeutic effect of ABMT are not clear [27, 28].

Other attention-based treatment approaches, for example, mindfulness-based interventions, have also reported positive results in people with obesity and/or BED. They train participants to attend to the present moment in a non-judgmental manner [29, 30]. This includes identifying internal cues of hunger/satiety and learning to control impulsive or automatic behaviours that could lead to overeating [31]. Systematic reviews and meta-analyses of randomised controlled trials (RCT) of mindfulness-based interventions in people with overweight/obesity have reported post-intervention reductions in binge eating episodes and impulsive eating (large effect sizes) and increases in physical activity (small to medium effects), although the effect of mindfulness on body weight is less clear. In addition, medium effect sizes have been reported regarding improvements on symptoms of depression and anxiety after such interventions [32, 33]. However, similarly to ABMT, there is little understanding of the underlying basis of the therapeutic effect of mindfulness-based interventions and, to date, no studies have measured the effects of these kind of interventions on food-related AB.

To our knowledge, there have been no RCTs of ABMT in people with obesity or BED. Investigating the clinical potential of ABMT and comparing it with a more established attention training (i.e. a short mindfulness training [MT], for details see below) will shed light on the most appropriate treatment. Furthermore, comparing different types of attention trainings (i.e. MT vs ABMT) in people with obesity will contribute to our understanding of maladaptive cognitive patterns (i.e. AB) related to food craving and overconsumption in this population.

### Aim and objectives

The aim of this trial is to assess the feasibility and acceptability of two different attention trainings (i.e. ABMT and MT) with a waiting-list control group in people who are overweight or obese and to obtain important information for the development a future large-scale RCT.

### Primary objective

- To establish the feasibility of conducting a large-scale RCT of attention trainings in patients who are overweight or obese, by assessing recruitment, attendance and retention rates.

### Secondary objectives

- To determine the best instruments for measuring outcomes in a future trial by examining the quality, completeness and variability in the data.
- To estimate the treatment effect sizes and standard deviations for outcome measures to inform the sample size calculation for a large-scale RCT.
- To determine whether patients who are overweight/obese view computer-based attention trainings as acceptable and credible.
- To obtain information about patients’ willingness to undergo random allocation to ABMT, MT or waiting list for eight weeks.
- To evaluate the relationship between AB and eating behaviour.
- To identify AB modification after training and its association with symptom change.
- To compare eating behaviour and weight change between conditions before and after training.
- To identify potential additional underlying mechanisms of attention trainings related to reward processing and self-regulatory processing using neurocognitive tasks.

## Methods

### Design

**Fig. 1:**
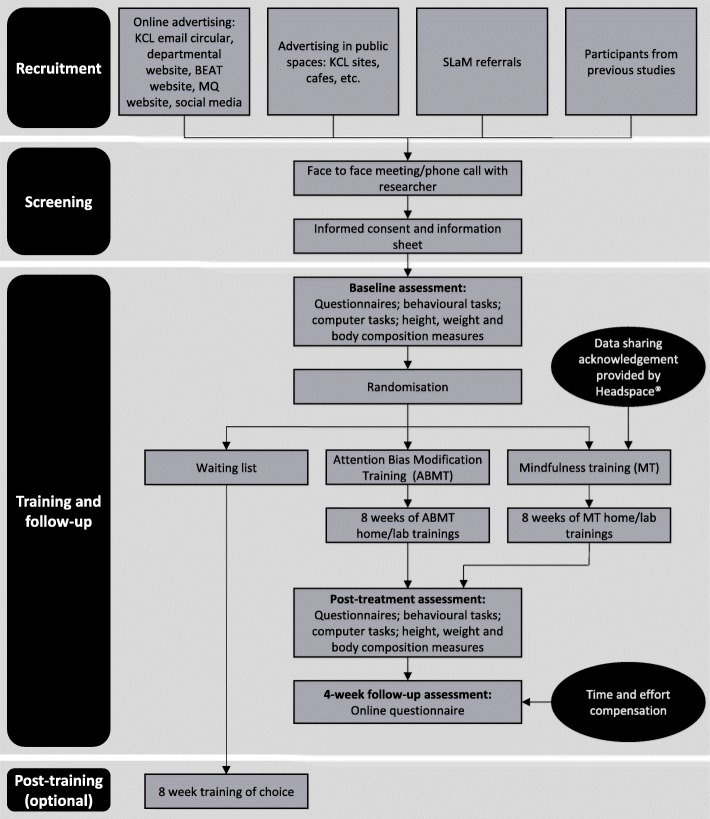
*Schematic diagram* of study procedures

This feasibility study is a three-parallel group, randomised, waiting-list control trial comparing ABMT versus MT for people who are overweight or obese. The protocol is outlined in Fig. 1 and details of the assessments timepoints are given in Table 1.

**Table 1:** Assessment timepoints

	Screen visit	Week 1 pre-assessment	Weeks 1–8	Week 8 post-assessment	Week 12 follow-up
Patient information and informed consent	X				
Data sharing Acknowledgement for Headspace® (when applicable)		X			
Eating Disorder Diagnostic Scale (EDDS)	X				
Structured clinical interview for DSM-V (SCID)	X				
Additional eligibility assessment	X				
Visual analogue scale (VAS) for hunger, craving, mood, stress and anxiety levels		X	X	X	
Eating Disorders Examination Questionnaire (EDEQ)		X		X	
Power of Food scale		X		X	X
Mindful Eating Questionnaire (MEQ)		X		X	X
Mindful Awareness and Attention Scale (MAAS)		X		X	
Depression, Anxiety and Stress Scale (DASS-21)		X		X	
State and Trait Anxiety Inventory (STAI)		X		X	
Weight, height and body composition		X		X	
Experimental tasks (Food-Choice task, Bogus taste test, Food-challenge task)		X		X	
Food-ANT		X		X	
Visual Probe Task		X		X	
ABMT/MT			X		
Acceptability questionnaire				X	
Eating habits, self-report body weight					X
Snack Diary (when applicable)			X		
Demographics		X			
Drink and food adherence		X	X	X	

### Setting

The study will be carried out at the Institute of Psychiatry, Psychology and Neuroscience (IoPPN) at King’s College London (KCL) and South London and Maudsley NHS Foundation Trust (SLaM), UK.

### Ethical approval and trial protocol

Ethical approval for the protocol of this study v.2.2 dated 18 October 2018 was obtained from London - City & East Research Ethics Committee (REC ref.: 18/LO/1683). The study is registered on the International Standard Randomised Controlled Trial Number (ISRCTN) registry (registration number: ISRCTN15745838).

### Participants and recruitment

Participants will be recruited via advertisement on the KCL circular mail and social media; posters with general information about the study will be placed on notice boards at various KCL sites and public places. Adverts will also be shown on different online platforms such as the Beat website (the UK’s national Eating Disorders Association), the research team’s departmental website and the MQ mental health charity website. In addition, suitable patients attending the Eating Disorders Unit at SLaM will be invited to take part in the study. Lastly, participants who have taken part in previous research and have consented to be contacted for other studies will be approached.

### Inclusion criteria

Male and female participants will be eligible if they: (1) are aged ≥ 18 years; (2) have a BMI ≥ 25 kg/m^2^; (3) are fluent in English; and (4) give written informed consent.

### Exclusion criteria

Participants will be excluded if they: (1) have a diagnosis of a current other major psychiatric disorder (e.g. major depression, major suicidality, substance dependence, psychosis) needing treatment in its own right; (2) have a past or present DSM-5 diagnosis of anorexia nervosa (AN), bulimia nervosa (BN) or Other Specified Feeding or Eating Disorder (OSFED); (3) have a diagnosis of diabetes mellitus; (4) have recently started psychotropic medication or increased the dose (i.e. within the previous two weeks); (5) take medication for weight loss; (6) are pregnant (either current or in the past six months); (7) have a regular, current or past, mindfulness meditation or yoga practice (defined as > 20 min, twice or more times per week during the past two months); or (8) have visual impairments that cannot be corrected with contact lenses or glasses.

### Sample size

The sample size of this feasibility study is based on standard suggestions considering 12 participants per arm as reasonable sample size for a feasibility trial mainly because estimates of the standard deviation for normally distributed variables tend to stabilise around this size [34]. Considering a sample size of *n* = 36 and assuming the attrition to follow-up rate is a = 0.25, we strive for a sample size of *n* = 45, i.e. 15 participants per group (using an attrition correction factor of 1/(1-a)).

### Randomisation

Participants will be allocated to either the ABMT, MT or waiting-list condition. Randomisation will be done by minimisation to control for BMI (obese/overweight) and gender (male/female). An independent researcher (not connected with the study) will perform the allocation using a computer programme for the generation of the random component. Allocation for balance between groups will be done manually and will be communicated via email to the researcher for each participant. Participants allocated to the waiting-list condition will be given the option to take the electronic version of the training of their choice (i.e. no randomisation process) once the waiting time is over.

### Intervention conditions

Participants assigned to either of the two intervention conditions will be asked to attend the Institute of Psychiatry, Psychology and Neuroscience eight times during an eight-week period (i.e. once/week). In addition, participants will be instructed to complete an Internet-based version of the allocated training every day for eight weeks at home (except for days when they have their weekly lab-based session).

For every lab-based training session, participants from both conditions will be asked to choose between three high-calorie snacks. The chosen snack will be available to them to activate craving and reward/self-regulation cognitions during training. Visual analogue scales (VAS) for hunger and craving for the chosen snack will be assessed before and after the trainings to measure any potential changes. In addition, to minimise variability in hunger and satiety, participants will be requested not to eat or drink anything (apart from water) 2 h before each training. Before this window, they will be advised to have a light meal. To establish compliance, drink and food adherence for each training session will be recorded.

Adherence to home sessions will be monitored via self-report (i.e. a training log) and through an output generated electronically at the time of each training. Other than these data on compliance, no further data from daily trainings will be collected.

### Attention bias modification training

The aim of the ABMT is to relatively implicitly train participants to ‘look towards’ low-calorie food and ‘look away’ from high-calorie food using a modified version of the anti-saccade task [16], while eye movements will be recorded to assess participants’ training accuracy. For home sessions, participants will be given a tablet (Asus ZenPad 10) with a website version of the ABMT training to use for a daily 10-min session. Participants allocated to this condition will be asked to record their daily accuracy rate on the training.

No side effects or risks of this type of training have been reported. However, because of the intense level of concentration required, participants could report fatigue. To minimise this, participants will be given small breaks (e.g. 2 min) between each block of the training (each training is formed by three blocks). We do not anticipate any other unintended effects of this training.

*ABMT training paradigm overview*: This version of a modified anti-saccade task consists of 360 trials in total. Of these, 180 require participants to look towards low-calorie foods and 180 trials require participants to look away from high-calorie foods. For all trial types, a black fixation point appears for 100 ms, followed by a red or blue fixation point for 500 ms. A blue point indicates that a pro-saccadic eye movement is required (i.e. looking towards the food picture appearing after the fixation point), whereas a red point requires an anti-saccadic eye movement (i.e. to direct the gaze to the opposite side of the screen to where the picture appears). A blank screen gap is inserted for 200 ms between fixation point and picture presentation to speed up subsequent reaction times (e.g. Kissler and Keil [35]) and then a pictorial stimulus (high- or low-caloric food picture) appears on either the left or the right side of the screen for 500 ms. Inter-trial interval is 1300 ms. Trials will be presented in three blocks of each 120 trials.

*ABMT paradigm trial types*: Low-calorie cues are always preceded by a blue dot (indicating that participants should perform a pro-saccade towards the stimulus) and high-calorie food cues are always preceded by a red dot (indicating that participants should perform an anti-saccade away from the stimulus).

*Stimuli:* Participants will view 30 low-calorie food and 30 visually matched high-calorie pictures per block. The position of each picture is counterbalanced for the presentation on the screen side: each picture presented once on the left and right sides of the screen, resulting in the overall 360 training trials (30 food stimuli + 30 non-food stimuli × 2 positions × 3 blocks). The order of trials will be randomised individually.

*Feedback:* Response latencies are recorded during the task to monitor accuracy and provide participants with feedback. For each block, numbers of correct responses are summed up and presented as a percentage score of correct performance to the participant (e.g. 70% correct performance).

### Mindfulness training

The app-based MT (provided by Headspace®) is 15 min long and guides participants through a combination of breathing exercises focusing on the present moment. Participants allocated to this condition will be requested to sign a data privacy acknowledgement to authorise the company to share information with the study researchers at KCL.

This type of intervention could trigger some anxiety in participants while they focus on the present moment. To minimise this, participants will be introduced to the basics of mindfulness before the intervention. Participants will be given the option to stop at any point during the training should they wish to do so due to any unintended effects of the training.

*Procedure:* Individuals allocated to the MT condition will be given free access to the Headspace® app for eight weeks. Participants will be instructed to download the app to their own mobile phones/electronic tablets and sign in with their newly created account. As a feature of the app, participants will be able to choose a time of day when they wish to receive a notification as a reminder of their daily meditation.

The MT is divided into two stages. The first four weeks focus on mindful eating meditation followed by four weeks focusing on overcoming cravings. Both stages (i.e. mindful eating and overcoming cravings) use a meditation technique called ‘noting’, which is an attempt to recognise those instances when the mind has wandered and identify the source of distraction, let it go and return to the object of focus (e.g. the breath).

Every training session (both lab-based and home sessions) will consist of 15 min of guided meditation. Both stages of the training (i.e. mindful eating and overcoming cravings) are divided into three competency levels: weeks 1–2 are the ‘Learn’ level; weeks 2–3 correspond to the ‘Practice’ level; and weeks 3–4-are for the ‘Master’ level.

### Waiting list

After baseline assessment, participants allocated to the waiting list condition will be asked to wait for eight weeks and, once they have completed the post-assessment after their wait, they can opt to receive the online version (i.e. daily home sessions only) of either ABMT or MT for eight weeks. After eight weeks of online training, participants will be asked to complete an electronic assessment including training adherence, current weight and a measure of eating disorder symptoms, eating habits and general mindfulness. No further data will be collected.

### Measures

#### Screening measures

- BMI (kg/m^2^);
- Eating Disorder Diagnostic Scale (EDDS) [36]: a 22-item questionnaire used to identify the presence of an eating disorder (i.e. AN, BN, BED);
- Structured clinical interview for DSM-V (SCID) [37]: this measure will be used to assess for any major psychiatric condition;
- Mindfulness practice: previous experience with mindfulness techniques (i.e. > 20 min, twice or more times per week during the past two months) will be assessed.

#### Within-session measures

Before and after each training session, participants in both conditions will complete a VAS measuring hunger and craving for the specific snack used in each session. VAS scales consist of a 10-cm line and participants are requested to indicate a degree or level of a specific emotion or behavioural urge ranging from ‘not at all’ to ‘severe’. After every lab-based training, participants will be asked to report what they ate or drank (apart from water) in the 2 h before the training.

#### Outcome measures

A range of outcome measures will be included with the aim of determining the most appropriate ones for a larger-scale RCT (based on effect sizes). The metric, timing and method of aggregation specifications for all outcome measures are described in the ‘Analyses’ section of this protocol. They are in accordance with Zarin et al. [38].

Clinical outcomes

- Eating behaviour-related measures: BMI will be calculated measuring body weight and height (kg/m^2^) and body composition (primarily body fat percentage) will be assessed using a bioelectrical impedance scale (InBody S10). The Eating Disorder Examination Questionnaire (EDE-Q) [39] assesses specific psychopathology of eating disorders using a 36-item self-report format. The Power of Food Scale (PFS) [40] evaluates the psychological impact of the modern ‘obesogenic’ environment. Dietary Recall over 24 h will be used to assess the quality of participants’ diet. VAS scales will be used to measure hunger and craving. In addition, VAS scales for cue-elicited food cravings will be administered after the Food Challenge Task [41] (short films created from Marks & Spencer’s adverts showing palatable foods). The bogus taste test [42] will measure food consumption of highly palatable food (i.e. crisps, chocolates and soft sweets) after asking participants to rate the food items according to smell, taste and attractiveness for 10 min. The difference in grams before and after the taste test will indicate the total food intake;
- Mindfulness and mindful eating-related outcomes: the Mindful Eating Questionnaire (MEQ) [43] is a 28-item questionnaire which will be used to assess awareness, distraction, disinhibition of eating and emotional and external eating. The Mindful Awareness and Attention Scale (MAAS) [44] is a 15-item scale to assess core characteristics of mindfulness;
- Other symptomatology: the Depression, Anxiety and Stress- Scale (DAAS-21) [45] is a 21-item self-report questionnaire which aims to evaluate mood, anxiety and stress levels over the previous week. The 20 items related to state-anxiety of the State and Trait Anxiety Inventory (STAI) [46] questionnaire will be used. VAS scales will also be used to measure current mood, stress and anxiety levels.

Neurocognitive outcomes

- The Food Attention Network Task (Food-ANT) will be used to assess three components of attention (i.e. orienting, alerting and executive function) using food (low- and high-calorie) versus non-food pictures (neutral items). This task was designed as reported by Hege et al. [47]. Briefly, trials will include a fixation cross, a cue to indicate where the target would appear, and a picture (i.e. the target). In 70% of the trials, participants will be presented with a single arrow pointing either to the left or the right side of the screen to indicate the position of the target. From these single arrow trials, 70% of the time the target will appear on the side where the arrow points (congruent) and 30% of the time the target will appear on the opposite side of the cue (incongruent).
  In 15% of the trials, a double arrow will appear indicating equal likelihood of the target appearing on either side of the screen and in the other 15% of the trials, the target will be presented without a cue (i.e. no arrow). In double arrow and no arrow trials, the target will appear equally on each side of the screen.

Reaction times (RTs) will be recorded and the different components of attention will be measured by subtracting RTs as follows:

- Alerting: a subtraction of the RTs of double arrow (bidirectional) cue condition from the RT of the no cue condition;
- Orienting: subtraction of RTs of the congruent directional cue from the RTs of the double arrow;
- Executive function: subtraction of the RTs of incongruent directional cue from congruent directional cue condition.

b) The visual Probe Task (VP) [22] will be used to assess visuo-spatial attention biases for food cues. Test–retest reliability of eye tracking indices (dwell time bias) and RT indices have recently been reported to be reliable measures of AB to food using this task [48]. During the VP, two pictorial stimuli matched for visual characteristics (i.e. one food and one non-food item) will be presented simultaneously on both sides of a computer screen followed by a dot appearing on either the left or the right side of the screen. Participants will be instructed to press the left or right arrow of the keyboard to indicate where the dot appeared. RTs will be recorded as an indirect measure of attention biases under the rationale that participants will be quicker to react to the dot appearing on the side of the picture they were attending to during the stimulus presentation. In addition, eye movements will be recorded as a direct measure of attention biases. The task will consist of two blocks of 60 trials each (120 trials in total). One block will present high-calorie food pictures against neutral items while the other will present low-calorie food pictures against neutral stimulus. The order of the blocks will be counterbalanced for each participant. The probability of the dot appearing behind the food picture or the neutral stimuli, as well as on the left or right side of the screen, will be equal (i.e. 50% on each side of the screen).
c) A modified version of the Food-Choice Task [49] adapted for eating disorders [50] will be used to assess for food preference (i.e. low-calorie vs high-calorie). In the first two blocks of the task, participants will be presented with 43 food pictures (25 low-fat and 18 high-fat). The instructions will be to rate the pictures of food according to tastiness and healthiness, respectively. Ratings will be measured using a 5-point scale: for the healthiness block, ratings will go from ‘unhealthy’ to ‘healthy’, whereas in the tastiness block, the scale will go from ‘bad’ to ‘good’. A food item rated as ‘neutral’ on both scales will be used as a reference food for the third block. During the third block or the ‘choice’ condition, participants will have to choose between their reference food and another food item imagining they would be presented with both options at the end of the study as a snack.

Acceptability and credibility

- A set of questions related to acceptability and credibility of the interventions will be provided. These questions will include items like ‘How credible did you find this training?’ and ‘How useful did you find this training?’ Questions related to perceived benefits and drawbacks of the intervention will also be recorded to inform any unintended effects of the trainings.

### Procedure

#### Screening

For participants recruited from general public spaces, study adverts will include the researcher’s contact details for participants to contact them if they are interested in taking part.

Potential participants from a clinical setting will be identified by their clinical care team and ask for consent to pass their details to the research team. Alternatively, patients will be given an information sheet with the researcher’s contact details and the option to contact them if they wish to.

Interested participants who either approach the researcher or are contacted by researcher will be invited to have a phone call for the researcher to answer any queries they may have. To check for eligibility, participants will be asked questions using the screening measures described above.

#### Baseline assessment

If participants are eligible and wish to take part, they will be sent an information sheet and a consent form. Once the consent form is signed, participants will be asked to attend the Institute of Psychiatry, Psychology and Neuroscience to complete a baseline assessment including all the outcome measures described above in addition to demographic and clinical information including age, gender, ethnicity, education, history of previous and current treatments, diet, and previous and current body weight. To control for hunger levels in the assessment session, participants will be asked to not eat or drink anything (apart from water) during the 2 h before the session. Adherence to this request will be measured by asking participants to list what they ate/drank in the last 2 h before the experiment.

#### Post-treatment assessment

The post-treatment assessment will be conducted after eight training sessions and will include all the measures used in the baseline assessment (apart from demographic information). In addition, to control for confounding variables, participants will be asked to answer a questionnaire about acceptability and credibility of the training as well as involvement in other weight-control intervention (including other research) during the eight weeks of training. A member of the research team will carry out assessment sessions with participants and will check outcome measures for completeness to ensure data quality.

Participants in the ABMT group will be asked to return the tablet after completion of the study.

#### Follow-up assessment

Four weeks after the last session (i.e. week 12), participants allocated to either ABMT or MT will be contacted via email and will be asked to complete an online version of the EDE-Q including a question on their current weight, the PFS and the MEQ.

For the waiting-list group, the above questionnaires will be sent to participants at the end of the eight weeks of online training in addition to a question related to training adherence and involvement in other weight-control programmes. Participants will be compensated with £50 for their time and effort after completion of the study.

### Withdrawal of participants

One of the main outcome measures of this study is participant retention. We have a number of strategies to encourage continued participation of individuals (e.g. weekly email reminders and monetary compensation at the end of study). Should a patient decide to withdraw from the study, reason for withdrawal will be recorded and listed in the final report/associated publications. No further data will be collected from participants who decide to withdraw from the study.

### Biological specimens

No biological specimens will be collected.

#### Blinding

Due to the heterogeneity of the study conditions, blinding of participants will not be possible. For resource reasons and given the feasibility nature of the study, we will also not be able to blind outcome assessors. It is our opinion that this is unlikely to introduce bias as main outcome measures are objective, biological or implicit measures, such as BMI, body composition and assessing of computerised tasks, e.g. reaction times. However, the data analyst will be blinded.

### Analyses

#### Feasibility

Feasibility of the trial will be related to recruitment as well as retention rates at both post-assessment and follow-up. Acceptability of both trainings and effect sizes of treatment outcomes will also inform the feasibility for a larger-scale RCT.

#### Clinical and cognitive outcomes

Analyses will follow the intention-to-treat principle. Quality, completeness and variability of the outcome measures will be determined by the use of descriptive statistical analyses and graphical methods. Group differences will be estimated using linear mixed effects regression models, controlling for the baseline level of the outcome and the strata variable used in the randomisation. The aim is not to determine significant group differences but to establish a suitably precise effect size for the primary outcome at the post-treatment assessment. This estimate will be used to guide the sample size of a future efficacy trial.

The size of the treatment effect on each outcome measure will be the difference in mean scores between conditions at post-treatment (eight weeks) and follow-up (12 weeks).

## Discussion

Preliminary evidence supports the role of attention processes such as attention biases to food on eating behaviour (e.g. food cravings) and suggests that novel attention-based interventions such as ABMT and mindfulness-based interventions have clinical potential [16, 18, 51–53]. However, the underlying mechanisms of these interventions are not well understood.

Here we described a protocol for a feasibility trial comparing two different attention trainings (i.e. ABMT and MT, our form of mindfulness-based intervention) for people with obesity. This study will be informative for the development of future larger-scale RCTs investigating the potential clinical effects of these types of attention trainings. In addition, results from this study will contribute to our understanding of the importance of attention processes in relation to food cues and eating behaviour. Specifically, the role of attention biases to food in obesity-related behaviours such as food craving and overconsumption of food.

While the aim of both ABMT and MT is to influence eating behaviour through the modification of attention processes, the focus of the training differs in terms of the aspect of attention that is being trained. ABMT has been developed to target AB to food in a relatively implicit way, presumably through strengthening attention control over attention allocation in the presence of food cues. In contrast, MT explicitly trains people to attend to the present moment (i.e. attention awareness) in a non-judgemental way. Investigating whether the clinical effects of both interventions are due to changes in AB to food and/or changes in self-regulation and reward processes will contribute to our understanding of the mechanistic components of ABMT and mindfulness-based interventions.

Potential challenges that this trial may face are related to recruitment and retention rates. Intervention length in studies using other mindfulness-based interventions in people with obesity is in the range of 4–24 weeks with good retention rates [52, 54–56]. Even though ABMT interventions in people with obesity have been most commonly limited to one session only [18, 57, 58], ABMT has been used for up to 10 sessions in studies for depression [59] and one feasibility study in people with obesity reported the use of ABMT for eight weeks [26]. However, undertaking a daily home practice for eight weeks of either training entails a high level of commitment that could hinder recruitment and lead to high rates of attrition.

In addition, participants allocated to the waiting-list condition might be discouraged due to the eight-week wait to receive a training. However, after the waiting period, they will have the option of choosing their preferred training. The opportunity to select an attention training as opposed to being randomly allocated to one, is likely to serve as motivation for waiting-list participants to continue as part of the trial.

In summary, novel treatment approaches in obesity are needed to achieve longer-term success in maintaining a healthy body weight and eating habits. The investigation of cognitive interventions which target maladaptive biases and potentially influence eating behaviour are a promising route. This study will evaluate the feasibility and acceptability of two different attention trainings in people with obesity and will shed light into their underpinning mechanisms and clinical potential. Results from this study will inform the design of larger RCTs aiming at investigating the efficacy of attention trainings and their potential to be implemented as adjunct treatment approaches for people with obesity.

## Trial status

Protocol version v.2.2 dated 18 October 2018. Participant recruitment and data collection for this study began on the 1 March 2019 and the expected date of completion is 1 March 2020.

## Supplementary information

**Additional file 1.** Participant Consent Form.

## Data Availability

The datasets used and/or analysed during the current study will be available from the corresponding author on reasonable request.

## Abbreviations

AB: Attention bias
ABMT: Attention Bias Modification Training
AN: Anorexia nervosa
BED: Binge eating disorder
BMI: Body mass index
BN: Bulimia nervosa
CBT: Cognitive Behavioural Therapy
DAAS-21: Depression, Anxiety and Stress Scale-21
EDDS: Eating Disorder Diagnostic Scale
EDEQ: Eating Disorders Examination Questionnaire
EDNOS: Eating disorder not otherwise specified
Food-ANT: Food-Attention Network Task
MAAS: Mindful Awareness and Attention Scale
MEQ: Mindful Eating Questionnaire
MT: Mindfulness training
PFS: Power of Food scale
RCT: Randomised controlled trial
SCID: Structured clinical interview for DSM-V
STAI: State and Trait Anxiety Inventory
VAS: Visual analogue scale
VP: Visual Probe Task
